# The Cannabinoid CB1 Antagonist TM38837 With Limited Penetrance to the Brain Shows Reduced Fear-Promoting Effects in Mice

**DOI:** 10.3389/fphar.2019.00207

**Published:** 2019-03-20

**Authors:** Vincenzo Micale, Filippo Drago, Pia K. Noerregaard, Christian E. Elling, Carsten T. Wotjak

**Affiliations:** ^1^ Research Group “Neuronal Plasticity”, Max Planck Institute of Psychiatry, Munich, Germany; ^2^ Department of Biomedical and Biotechnological Sciences, Section of Pharmacology, University of Catania, Catania, Italy; ^3^ National Institute Mental Health, Klecany, Czechia; ^4^ 7TM Pharma A/S, Hørsholm, Denmark

**Keywords:** cannabinoid CB1 receptor, rimonabant, peripheral CB1 receptor antagonist, TM38837, fear conditioning

## Abstract

Rimonabant was the first selective CB1 antagonist/inverse agonist introduced into clinical practice to treat obesity and metabolic-related disorders. It was withdrawn from market due to the notably increased rates of psychiatric side effects. We have evaluated TM38837, a novel, largely peripherally restricted CB1 antagonist, in terms of fear-promoting consequences of systemic vs. intracerebral injections. Different groups of male C57BL/6 N mice underwent auditory fear conditioning, followed by re-exposure to the tone. Mice were treated *per os* (p.o.) with TM38837 (10, 30, or 100 mg/kg), rimonabant (10 mg/kg; a brain penetrating CB1 antagonist/inverse agonist which served as a positive control), or vehicle, 2 h prior the tone presentation. Only the high dose of TM38837 (100 mg/kg) induced a significant increase in freezing behavior, similar to that induced by rimonabant (10 mg/kg) (*p* < 0.001). If injected into the brain both TM38837 (10 or 30 μg/mouse) and rimonabant (1 or 10 μg/mouse) caused a sustained fear response to the tone, which was more pronounced after rimonabant treatment. Taken together, TM38837 was at least one order of magnitude less effective in promoting fear responses than rimonabant. Given the equipotency of the two CB1 antagonists with regard to weight loss and metabolic syndrome-like symptoms in rodent obesity models, our results point to a critical dose range in which TM3887 might be beneficial for indications such as obesity and metabolic disorders with limited risk of fear-promoting effects.

## Introduction

Based on the animal and clinical studies showing that a pathological overactivation of the endocannabinoid transmission through the cannabinoid CB1 receptor contributes to obesity (for review, see Di Marzo et al., 2011), the CB1 antagonist/inverse agonist rimonabant (SR141716A, Rinaldi-Carmona et al., 1994) was the first compound introduced into clinical practice as an antiobesity agent in several countries (Rimonabant in Obesity: RIO studies) (Christopoulou and Kiortsis, 2011). However, enthusiasm for such agent has waned as a result of the withdrawal from the market due to the higher incidence in treated patients as compared to placebo controls of psychiatric side effects such as mood symptoms, anxiety, and suicidal tendencies (Van Gaal et al., 2005; Christensen et al., 2007). Despite this experience, there is still interest in the development of CB1 antagonism as a pharmacological tool for the treatment of metabolic disorders, however, with a better safety profile (Le Foll et al., 2009; Janero et al., 2011; Ward and Raffa, 2011; Kirilly et al., 2012). In this context, two main alternatives are currently discussed: (1) the use of CB1 neutral antagonist, such as AM4113, NESS0327, or AM6545, instead of the CB1 receptor antagonists/inverse agonists (e.g., rimonabant), which have recently shown efficacy to reduce body weight and food intake in rodents with less unwanted side effects than rimonabant (Sink et al., 2008, 2010a,b; Meye et al., 2013; Gueye et al., 2016) and (2) the use of peripherally directed CB1 inverse agonist/antagonist, which revealed promising preclinical results to reduce body weight (Tam et al., 2012, 2018; Chorvat, 2013; Sharma et al., 2018). Among them, TM38837 was shown to induce a significant weight loss in obese mice similarly to rimonabant (Noerregaard et al., 2010), with no clear central nervous system (CNS) effects and a potential favorable side effects profile (Klumpers et al., 2013), possibly because of reduced brain CB1 receptor occupancy (Takano et al., 2014). Since previous data have consistently shown that genetic or pharmacological blockade of intracerebral CB1 receptors leads to sustained conditioned fear in rodents (for reviews see Riebe et al., 2012; Micale et al., 2013), a CB1 antagonist with confined actions in the periphery is expected to preserve its beneficial functions on several aspects of the metabolic syndrome, without exerting its psychopathological side effects. Thus, the peripheral-restricted CB1 antagonist TM38837 provides a very interesting tool to answer this question.

Based on the above premises, this study was undertaken to assess the effects of systemic (*per os – p.o.*) and local (*intracerebroventricular – icv*) treatment with the cannabinoid CB1 antagonist TM38837 on expression of conditioned fear in mice. If CB1 controls fear adaptation primarily *via* cortical glutamatergic neurons (Kamprath et al., 2009), it is expected that a CB1 antagonist (such as rimonabant) used here as a positive control with free access to the brain will inhibit fear adaptation (Marsicano et al., 2002; Plendl and Wotjak, 2010; Höfelmann et al., 2013). Systemic administration of an antagonist with restricted access to the brain such as the CB1 antagonist TM38837, in contrast, is expected to leave fear adaptation largely unaffected.

## Materials and Methods

### Animals

Male C57BL/6 N (B6N, 7–8 weeks old, purchased from Charles River) mice (*n* = 9–13 per group) were single housed in type 2 Macrolon cages and maintained in standard conditions with food and water *ad libitum* under a 12 h inversed light-dark cycle (lights off at 9 a.m.) for at least 14 days before starting the experiments. All behavioral experiments were performed between 9:30 a.m. and 5 p.m. (i.e., during the active phase of the animals). Note that the final sample size used for analysis was smaller due to heavy fighting in the home cage before separation, failures to cope with p.o. treatment by gavage (irrespective of the compounds), or escape during the experiment. All behavioral tests took place in an experimental room with the same light-dark cycle and environmental conditions (i.e., humidity, temperature) as in the housing facility. All experimental procedures were approved by the Government of Upper Bavaria (55.2.1.54-2532-44-09; 55.2.1.54-2532-141-12). All experiments were carried out according to the European Community Council Directive 2010/63/EEC, and efforts have been made to minimize animal suffering and reduce the number of animals used.

### Behavioral Procedure

#### Fear Conditioning

The set-up has been described and displayed in detail before (Kamprath and Wotjak, 2004; Direnberger et al., 2012; Yen et al., 2012; Llorente-Berzal et al., 2015). Briefly, mice were placed in the conditioning context (chamber) (d0). Three minutes later, a tone (80 dB, 9 kHz sine wave, 10 ms rising and falling time) was presented to the animals for 20 s that coterminated with a 2-s scrambled electric foot shock of 0.7 mA. Mice were returned to their home cages 60 s later.

#### Tone Re-exposure

Mice were placed in test context, which differed from the conditioning context in material, shape, surface texture, and odor of the cleaning solution (cylinder; Kamprath and Wotjak, 2004). After an initial 3 min of habituation, mice were confronted with a permanent 180 s tone (80 dB, 9 kHz, sine wave). Mice were returned to their home cage 60 s after the end of the exposure protocol. Tone re-exposure was started 24 h after conditioning and performed at d1, d2, d3, and d10 after the conditioning (Plendl and Wotjak, 2010; Llorente-Berzal et al., 2015).

#### Behavioral Analysis

The behavior of the mice was videotaped and scored off-line by a trained observer who was blind to the animals’ treatment by typing preset keys on a keyboard (EVENTLOG, Robert Henderson, 1986). Freezing was defined as the absence of all movements, except for those related to respiration.

### Drugs and Experimental Design

Two different experiments were performed. In *Experiment 1*, rimonabant (Kd~0.61 nM; Rinaldi-Carmona et al., 1996) (RIM: 10 mg/kg, National Institute of Mental Health Chemical Synthesis and Drug Supply Program), TM38837 (Kd~16 nM; Noerregaard et al., 2010) (10, 30, or 100 mg/kg, 7TM PHARMA), or vehicle (VHC: 0.1% Tween 80 and 1% hydroxypropyl methylcellulose, Sigma) were administered *per os* (p.o.) in a volume of 5 ml/kg, 2 h prior the tone re-exposure (days 1–3). On day 10, all the mice were treated with vehicle (VHC) 2 h prior the exposure to a 3-min tone. The dose of rimonabant (10 mg/kg) was selected based on a dose-response curves experiment, where an additional group of mice was treated with rimonabant (RIM 3 mg/kg, s.c.) as a positive control (Plendl and Wotjak, 2010; Terzian et al., 2014), 1 h prior to exposure to the 3-min tone. On day 11, four groups of mice (*n* = 5–6 per group) were treated with TM38837 (TM: 10, 30 or 100 mg/kg, p.o.) or rimonabant (RIM: 10 mg/kg, p.o.) and, 2 h later, were decapitated after short isoflurane anesthesia, and trunk blood was collected in pre-chilled EDTA tubes (KABE, Nümbrecht-Elsenroth, Germany). The samples were centrifuged at 1500 g for 10 min at 4°C. The entire resultant plasma obtained was transferred to suitably labeled polypropylene tubes and stored upright at −20°C for subsequent measurement of plasma levels.

In ***Experiment 2***, rimonabant and TM38837 were dissolved in vehicle solution (10% DMSO and 10% Cremophor EL in saline) (Sigma). The compounds were administered intracerebroventricularly (icv) in a volume of 2.0 μl/mouse. Different groups of mice were treated icv with TM38837 (TM: 10 or 30 μg/mouse), rimonabant (RIM: 1 μg/mouse), or vehicle (VHC) 30 min prior to re-exposure to the tone (days 1–3). On day 10, all mice were treated with vehicle (VHC) 30 min prior the tone re-exposure. The dose of rimonabant (1 μg/mouse) was selected on the basis of a dose-response experiment. For all groups, injections were given under light isoflurane (Forene®; Abbott, Wiesbaden, Germany) anesthesia to avoid differences in coping with the stressful injection procedure. The injection cannula protruded the guide cannula by 1 mm.

### Surgery

Following preoperative analgesia with Meloxicam (Metacam®, Boehringer-Ingelheim, Ingelheim, Germany; 0.5 mg/kg in 0.9% saline, s.c.), mice were deeply anesthetized with isoflurane and fixed to a stereotaxic frame (TSE-Systems, Heidelberg, Germany). Body temperature was kept constant at 36°C by a feedback-controlled heating pad. Two holes were drilled into the skull in order to insert an anchoring screw and a guide cannulae (manufactured from injection cannulae, 23 G; Braun-Melsungen, Melsungen, Germany). The guide cannula was implanted as follows (0.3 mm posterior to Bregma, 1.0 mm laterally from midline, 1.2 mm beneath the surface of the skull). Fixation was achieved with dental cement (Dual Cement; Ivoclar, Schaan, Liechtenstein). The wound was disinfected with Braunoderm® and closed with sutures. Post surgery, mice received Meloxicam (0.5 mg/kg i.p.) for 3 days and were allowed to recover for 10–14 days before the experiment. The recovery process was monitored daily by visual inspection. The injection cannula extended the guide cannula by 1 mm, thus reaching into the lateral ventricle.

### Analysis of Cannula Placement

At completion of behavioral testing, all mice of Experiment 2 were anesthetized with a ketamine/xylazine mixture and injected with 1.0 μl of Cresyl Violet icv in order to verify the injection sites. Brains were removed 20 min later. Histological examinations revealed particles of the ink in the lateral and third ventricles, but not in the brain parenchyma. We have used only data obtained from mice exhibiting a correct insertion at histological examination.

### Statistical Analysis

Freezing behavior was analyzed in 20-s interval or averaged over the entire tone presentation (180 s) and expressed as a percentage of the respective analysis interval. Data were analyzed using one-way ANOVA (total freezing) or 2-way ANOVA for repeated measures (development of the freezing response over the course of tone presentation) by means of SPSS 17.0 (Chicago, IL, USA) and GraphPad Prism 5.0 (GraphPad Software Inc., San Diego, CA, USA). Newman-Keuls test was used as post-hoc test, if appropriate. Data are presented as mean ± SEM. Statistical significance was accepted if *p* < 0.05.

## Results

This study was based on the comparison of fear-promoting effects of CB1 receptor antagonists with limited (TM38837 – TM) vs. unrestricted (rimonabant – RIM) penetrance into the brain.

### Experiment 1: Systemic Antagonist Treatment

**Figure 1:**
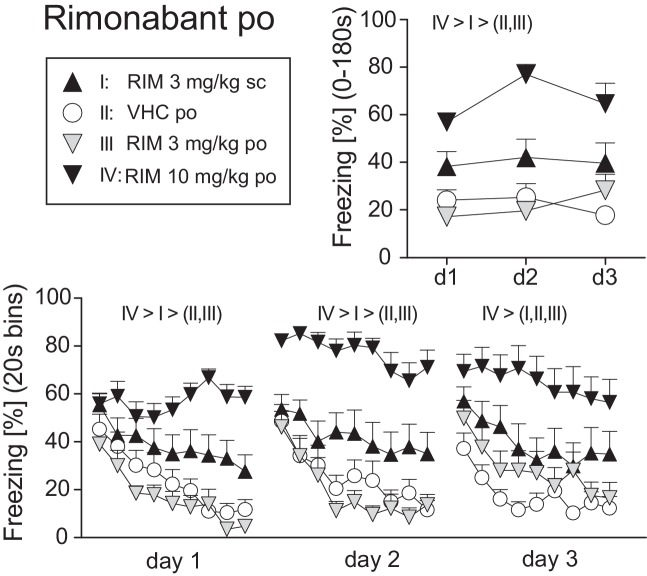
Effects of systemic rimonabant treatment on the expression of auditory-cued fear memory. Freezing responses (mean ± S.E.M.) of the animals after *per os* (RIM: 0, 3, or 10 mg/kg) or *subcutaneous* rimonabant (RIM: 3 mg/kg) treatment before re-exposure to the 3 min tone at days 1–3 after conditioning (day 0) averaged over the entire 180 s tone presentation (upper right) or in 20-s interval (lower panel). *n* = 11–13 mice per group. Data were analyzed by two-way ANOVAs for repeated measurements. Newman-Keuls post-hoc comparisons for the main effects *Treatment* are indicated above the graphs. For the matter of clarity, we do not report post-hoc results in case of factorial interactions.

Before starting with TM38837 (TM) treatment, we defined the dose of rimonabant (RIM), which causes sustained fear upon *per os* (*p.o.*) treatment compared to subcutaneous (s.c.) treatment as positive control (Experiment 1). Mice underwent auditory fear conditioning (day 0) followed by random assignment to one out of four groups (RIM 3 mg/kg s.c., VHC p.o., RIM 3 mg/kg p.o., or RIM 10 mg/kg p.o.). Mice were treated on three consecutive days 2 h (p.o.) or 1 h (s.c.) before re-exposure to the conditioned tone for 3 min. Analysis of the total freezing responses to the tone shown at the three consecutive days revealed that 10 mg/kg p.o. and 3 mg/kg s.c., but not 3 mg/kg RIM p.o., caused increased fear, compared to vehicle-treated controls, with 10 mg/kg being most effective [Treatment: F_3,43_ = 22.08, *p* < 0.0001; Treatment × Day: F_6,86_ = 1.845, *p* = 0.099; 2-way ANOVA (treatment, day) for repeated measures (day); Figure 1]. If analyzed in 20-s interval, all mice showed the same initial freezing response at day 1. However, whereas mice treated with vehicle or 3 mg/kg RIM p.o. showed a rapidly waning freezing response, treatment with 3 mg/kg s.c. (= positive control) and 10 mg/kg p.o. led to sustained fear (Treatment: F_3,43_ = 15.55, *p* < 0.0001; Treatment × Time: F_24,344_ = 3.533, *p* < 0.0001). Treatment at days 2 (Treatment: F_3,43_ = 20.92, *p* < 0.0001; Treatment × Time: F_24,344_ = 1.795, *p* < 0.05) and 3 (Treatment: F_3,43_ = 8.584, *p* < 0.0005; Treatment × Time: F_24,344_ = 1.502, *p* = 0.06) revealed essentially the same effects except for the increase in initial freezing observed in mice treated with RIM 10 mg/kg p.o. (Figure 1).

**Figure 2:**
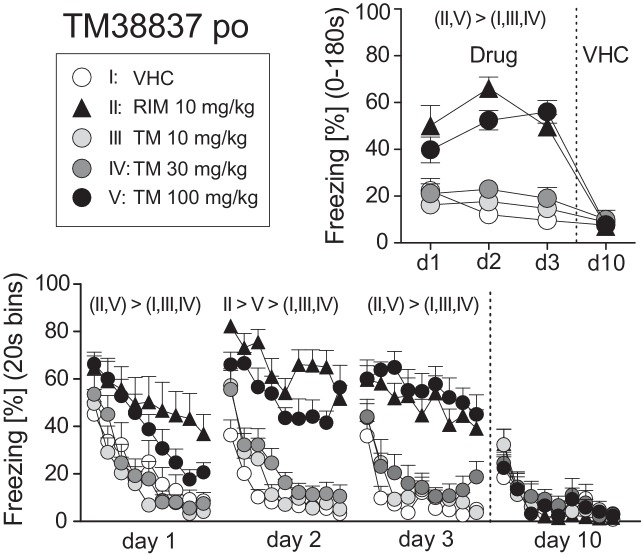
Effects of systemic treatment with the peripheral-restricted CB1 antagonist TM38837 on the expression of auditory-cued fear memory. Freezing responses (mean ± S.E.M.) of the animals after *per os* treatment with TM38837 (TM: 10, 30 or 100 mg/kg), rimonabant (RIM: 10 mg/kg), or vehicle (VHC) before re-exposure to the 3 min tone at days 1–3 after conditioning (day 0) averaged over the entire 180 s tone presentation (upper right) or in 20-s interval (lower panel). *n* = 9 mice per group. For further details, see Figure 1.

On basis of this dose-response study, we selected 10 mg/kg RIM p.o. as the reference dose for Experiment 2. New cohorts of mice underwent auditory fear conditioning (day 0) followed by re-exposure to the conditioned tone at days 1, 2, 3, and 10. At days 1–3, mice were treated with VHC, RIM 10 mg/kg (positive control), or TM 10, 30, or 100 mg/kg p.o. 2 h before tone presentation; at day 10, all mice received vehicle. Analysis of the total freezing responses revealed that 10 mg/kg RIM and 100 mg/kg TM caused a significant increase in conditioned fear, whereas 30 and 10 mg/kg TM were indistinguishable from vehicle treatment. These treatment effects were abolished at day 10 when all mice received vehicle (Treatment × Day: F_12,120_ = 8.726; *p* < 0.0001; Figure 2). Analysis in 20 s bins separately for days 1–3 confirmed the sustained freezing responses in mice treated with 100 mg/kg TM p.o. or 10 mg/kg RIM p.o., as compared to the other groups (Treatment: F_4,40_ > 6.454, *p* < 0.0005; Treatment × Time: F_32,320_ = 1.649, *p* < 0.05; Figure 2). Importantly, in no case, there were significant differences between mice treated with vehicle and TM 10 or TM 30 mg/kg. Also, drug treatment had no general effects on exploratory behavior, as exemplarily assessed by measuring freezing/immobility during the 20 s preceding the first tone presentation at day 1 (VHC: 18.1 ± 4.1%, RIM10: 17.8 ± 3.2%, TM10: 20.3 ± 3.8%, TM30: 20.3 ± 4.2%, TM100: 17.5 ± 3.8%). The plasma drug concentrations (mean ± SEM) 2 h post treatment were as follows: RIM 10 mg/kg = 139 ± 12 nM; TM 10mg/kg = 9,955 ± 1,325 nM; TM 30 mg/kg 113,574 ± 14,129 nM; TM 100 mg/kg = 178,479 ± 11,977 nM.

### Experiment 2: Intracerebral Antagonist Treatment

**Figure 3:**
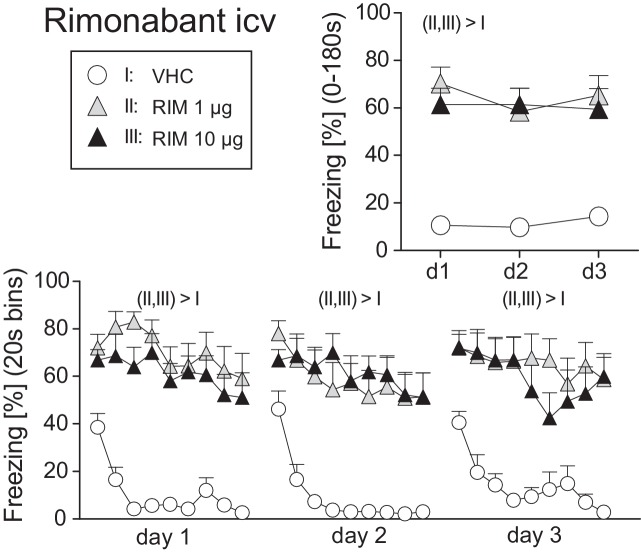
Effects of intracerebroventricular rimonabant treatment on fear memory in mice. Freezing responses (mean ± S.E.M.) of the animals after intracerebroventricular (icv) rimonabant (RIM: 1 or 10 μg/mouse) or vehicle (VHC) treatment averaged over the entire 180s tone presentation (upper right) or in 20s intervals (lower panel). *n* = 11-12 mice per group. For further details see Figure 1.

In order to include RIM as a positive control for intracerebral TM treatment, we first assessed the efficacy of different doses of RIM (1 vs. 10 μg) administered intracerebroventricularly (icv) on the expression of auditory-cued fear memory at days 1–3 after conditioning (day 0). Analysis of the total freezing responses revealed increased freezing following treatment with 1 and 10 μg RIM compared to vehicle controls, with no differences between the two doses (Treatment: F_2,31_ = 19.98, *p* < 0.0001; Treatment × Day: F_4,62_ = 1.10, *p* = 0.3625; Figure 3). If analyzed in 20-s interval, mice treated with vehicle showed a rapidly waning freezing response, whereas icv treatment with 1 or 10 μg lead to sustained fear. This became evident at all three experimental days (Treatment × Time: F_16,248_ > 1.8350, *p* < 0.05; Figure 3).

**Figure 4:**
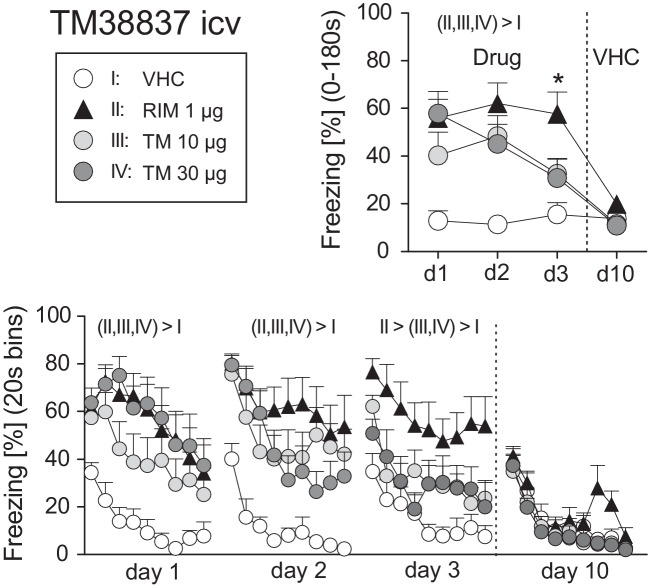
Effects of intracerebroventricular treatment with the peripheral restricted CB1 antagonist TM38837 on the expression of auditory-cued fear memory. Freezing responses (mean ± S.E.M.) of the animals after *intracerebroventricular* treatment with TM38837 (TM: 10 or 30 μg/mouse), rimonabant (RIM: 1 μg/mouse) or vehicle (VHC) averaged over the entire 180s tone presentation (upper right) or in 20s intervals (lower panel). *n* = 10 mice per group. *p < 0.05 vs. I, III and IV. For further details see Figure 1.

On basis of this dose-response study, we selected 1 μg RIM as the reference dose for comparisons with icv TM (10 or 30 μg). New groups of mice underwent surgery and fear conditioning and were treated before expression of auditory-cued fear memory at days 1–3 after conditioning (day 0); at day 10, all mice received vehicle. Analysis of the total freezing responses revealed a significant Treatment × Day interaction (F_9,108_ = 4.086, *p* < 0.0005). Post-hoc analyses confirmed that vehicle-treated controls froze significantly less than all other treatment groups at days 1–3, but not day 10. In addition, mice treated with 10 or 30 μg TM showed significantly less freezing than mice treated with 1 μg RIM at day 3 (Figure 4). These findings were confirmed if we compared the development of freezing over the course of the tone presentation separately for days 1–3 (Treatment: F_3,36_ > 5.588, *p* < 0.005; Figure 4).

## Discussion

In the present study, we demonstrate that the peripherally restricted CB1 antagonist TM38837 elicited fear-promoting effects following systemic treatment only at a dose 10 times higher than rimonabant. More specifically, the dose of 100 mg/kg p.o. appeared to be as potent as rimonabant (10 mg/kg, p.o.) to induce a sustained fear response upon recall of auditory-cued fear memory. Mice treated with lower doses of TM38837 (10 and 30 mg/kg, p.o.) were indistinguishable from the vehicle-treated control group. This observation is in accordance with the negligible access of TM38837 to the brain at therapeutic effective doses in mice (Noerregaard et al., 2010), the low brain CB1 receptor occupancy in nonhuman primates (Takano et al., 2014), and the approx. 10 times stronger rimonabant affinity to the CB1 receptors as compared to TM38837. Interestingly, TM38837 exerts its beneficial effects in animal models of metabolic diseases at similar doses as rimonabant (Noerregaard et al., 2010). Furthermore, the higher plasma concentration of TM38837 as compared to rimonabant level could be due as recently described to the low clearance and long terminal half-life of the peripherally restricted CB1 receptor antagonist (Klumpers et al., 2013) and its limited penetrance through biological membranes into different tissues.

TM38837 is belonging to the subclass of peripherally restricted CB1 antagonists. It was developed as a neutral antagonist with highly limited penetrance to the brain in order to minimize or prevent CNS adverse reactions while preserving potential antiobesity effects (Ward and Raffa, 2011; Chorvat, 2013). With the discovery of the endocannabinoid system, blockade of CB1 receptors became a preferred drug target. In line with this strategy, a particular emphasis has been on the antiobesity potential of prototypical selective CB1 receptor antagonist/inverse agonist rimonabant (Rinaldi-Carmona et al., 1994), which was discontinued, however, once its use was associated with psychiatric side effects (Van Gaal et al., 2005; Christensen et al., 2007; Moreira and Crippa, 2009; Christopoulou and Kiortsis, 2011; Micale et al., 2015). Thus, orally bioavailable CB1 receptor antagonists with molecular properties that limit their penetration across the blood-brain barrier and restrict their CNS access may reduce obesity-associated cardiometabolic risk with improved safety over rimonabant (Shrestha et al., 2018). This concept is based on the fact that CB1 receptors at peripheral sites (e.g., adipocytes or hepatocytes) could decisively influence energy expenditure and body fat storage/disposition, since visceral fat accumulation has been correlated with peripheral endocannabinoid system hyperactivity in human obesity (Cota et al., 2003; Silvestri et al., 2011; Bellocchio et al., 2013).

Our results confirm that the CB1 receptor antagonist/inverse agonist rimonabant following systemic administration is able to inhibit fear adaptation, further supporting the concept that pharmacological (Marsicano et al., 2002; Kamprath et al., 2006, 2009; Plendl and Wotjak, 2010; Höfelmann et al., 2013; Llorente-Berzal et al., 2015) as well as genetic (Haller et al., 2002; Marsicano et al., 2002; Jacob et al., 2009, 2012; Terzian et al., 2011; Metna-Laurent et al., 2012; Rey et al., 2012; Micale et al., 2017) inactivation of CB1 signaling exerts fear promoting/anxiogenic effects (for review, see Riebe et al., 2012). However, in our study, only the highest dose of TM38837 increased fear response, which was far more than the dose used to ameliorate metabolic symptoms (10 mg/kg; Noerregaard et al., 2010).

To analyze whether the difference in fear expression after systemic treatment could be attributed to the negligible access of TM38837 to the brain as compared to the good brain penetration of rimonabant, we injected the two compounds directly into the brain. Intracerebroventricular administration of both CB1 antagonists increased the fear response, even though rimonabant elicited a more pronounced and prolonged fear response as compared to TM38837. In fact, the effects of TM38837 were observed at higher doses and started to wane upon repeated treatment. This might be ascribed, at least in part, to the lower affinity to CB1 receptors compared to rimonabant (Takano et al., 2014).

In conclusion, TM38837 and rimonabant showed great similarity in their potential therapeutic effects against obesity and metabolic disorders (Noerregaard et al., 2010; Ward and Raffa, 2011; Kirilly et al., 2012; Shrestha et al., 2018). Our findings suggest that they could differ on potentially harmful effects, supporting a favorable prognosis for the absence of adverse side effects in case of chronic systemic treatment with the peripheral CB1 antagonist. Although further preclinical studies and controlled clinical studies are necessary to assess the efficacy and the safety profile of TM38837, these findings correspond well with the alternative approach in the treatment of obesity, which could be represented by the use of peripheral or neutral CB1 antagonists, lacking many of the adverse events associated with CB1 inverse agonist (Shrestha et al., 2018; Tam et al., 2018). Nevertheless, TM3887 is not devoid of fear-promoting effects, even though at 10 times higher concentrations than rimonabant, both after systemic and intracerebral injection.

Limitations of the study: Our study has a number of limitations which have to be considered. First, given the differences in receptor affinity, fear-promoting effects of TM38837 may become evident at higher concentrations (as shown in the present manuscript). In this context, it is of importance to define a low dose of TM38837 treatment, which still exerts its beneficial effects on metabolic syndrome while avoiding adverse effects on fear expression. Interestingly, plasma concentrations of TM38837 were orders of magnitude higher than that of the lipophilic rimonabant, which may favor peripheral effects. Second, our conclusions rely on a single behavioral readout (i.e., expression of conditioned fear), which might be additionally “contaminated” by unspecific effects in particular of rimonabant, even at lower doses (3 mg/kg), on locomotor activity (e.g., Llorente-Berzal et al., 2015). Therefore, future studies have to significantly broaden the number of behavioral measures of “discomfort,” including anxiety-related behavior and hormonal stress responses to unequivocally demonstrate the superiority of TM38837 compared to rimonabant in terms of potential side effects on emotional regulation.

## Author Contributions

VM has designed the study, performed the experiments, analyzed the data, and written the draft of the manuscript. FD has participated and contributed to the experimental design and data interpretation. CE has provided the new compound and discussed the data. CW has designed the study, supervised the experiments, contributed to data analysis, and participated in manuscript preparation. PN has participated in developing the concept and designing parts of the experiments. In addition, she has contributed data about pharmacokinetics and locomotor activity.

### Conflict of Interest Statement

VM, FD, and CW declare that they have no conflicting interests and that 7TM Pharma A/S had no influence on design, performance, analysis, and interpretation of the findings. CE is an employee of 7TM Pharma A/S, which provided drugs and financial support.

The remaining author declares that the research was conducted in the absence of any commercial or financial relationships that could be construed as a potential conflict of interest.
